# Trichotillomania in a Male Patient With Depression: A Case Report

**DOI:** 10.7759/cureus.73362

**Published:** 2024-11-10

**Authors:** Konstantinos Kontoangelos, Theodora Zafeiropoulou, Argyro Anna Karamparpa, Evangelos Daskalakis, Alexander Stratigos

**Affiliations:** 1 First Psychiatry Department, Eginition Hospital, National and Kapodistrian University of Athens, Athens, GRC; 2 First Dermatology Department, Andreas Syggros Hospital for Skin Diseases, National and Kapodistrian University of Athens, Athens, GRC

**Keywords:** depression, personality, psychodermatology, skin disorder, trichotillomania

## Abstract

Trichotillomania is a disorder of chronic hair pulling classified as an impulse control disorder that causes essential stress and leads to difficulties in functionality and severe alopecia. This is the case of a 43-year-old male without a serious medical history who was hospitalized with a large ulcerated plaque with smooth margins covering almost the entire occipital area. He received antibiotic and antidepressant treatment, after which he appeared to be improving. Psychotherapy with behavioral techniques and medication treatment may be effective. The case of a patient with trichotillomania is presented as psychiatric comorbidities on treatment outcomes.

## Introduction

Trichotillomania, also known as a hair-pulling disorder, is characterized by the constant pulling of hair mainly on the scalp and causes scarring hair loss. This condition is exacerbated by stress and can cause significant distress, shame, and low self-esteem [1]. Hair pulling is characterized as an impulse control disorder with severe compulsion, creating dysfunction and severe, noticeable alopecia, most commonly in scalp hair [2]. Due to the constant handling of the hair, patients may experience carpal tunnel syndrome, microbial infections, and scalp discoloration [3]. The lack of clear epidemiological data on trichotillomania also leads to inconsistent data on the gender distribution of trichotillomania in the general population, as most studies that are based on clinical samples suggest a female preponderance with a female-to-male ratio of 4:1 [4]. Specific neurotransmitters and markers of inflammation have been associated with the etiology of trichotillomania, and the literature reports that pre-treatment clinical symptom severity in patients with trichotillomania was associated with glutamate levels in certain areas of the brain, particularly in the anterior cingulate cortex [5]. Severe trichotillomania is associated with more extensive and pronounced perfusion abnormalities on single photon emission computed tomography (SPECT) scans, which have shown abnormalities in the parietal, frontal, and occipital areas [6]. In another study, blood oxidant levels were measured, and stress-related biomarkers in trichotillomania patients such as glutathione values were found to be reduced in comparison with the normal values. Also, low glutathione levels have been correlated with significant clinical burden [7]. Over 45% of children with trichotillomania endorsed depressive symptoms and 40% endorsed anxiety symptoms in excess of one standard deviation (SD) above published community norms. More remarkably, 25% of our sample reported depressive and 20% reported anxiety symptoms [8]. Trichotillomania must be differentiated from alopecia areata and traction alopecia. Trichoscopy reveals disorders due to the stretching and breakage of hair shafts by presenting trichoscopic signs such as black dots, yellow dots, and broken hair shafts of different lengths [9]. This is a case report of a 43-year-old male who was hospitalized with severe trichotillomania.

## Case presentation

**Figure 1:**
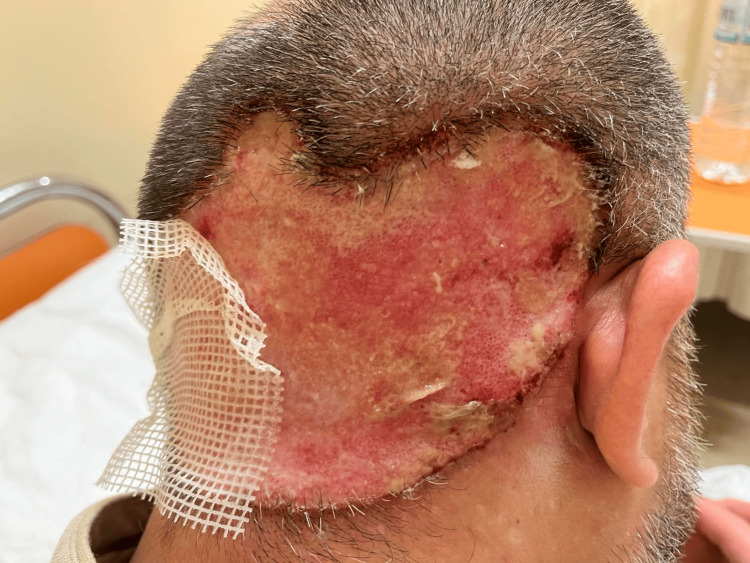
Back of the head during the first day of hospitalization

**Figure 2:**
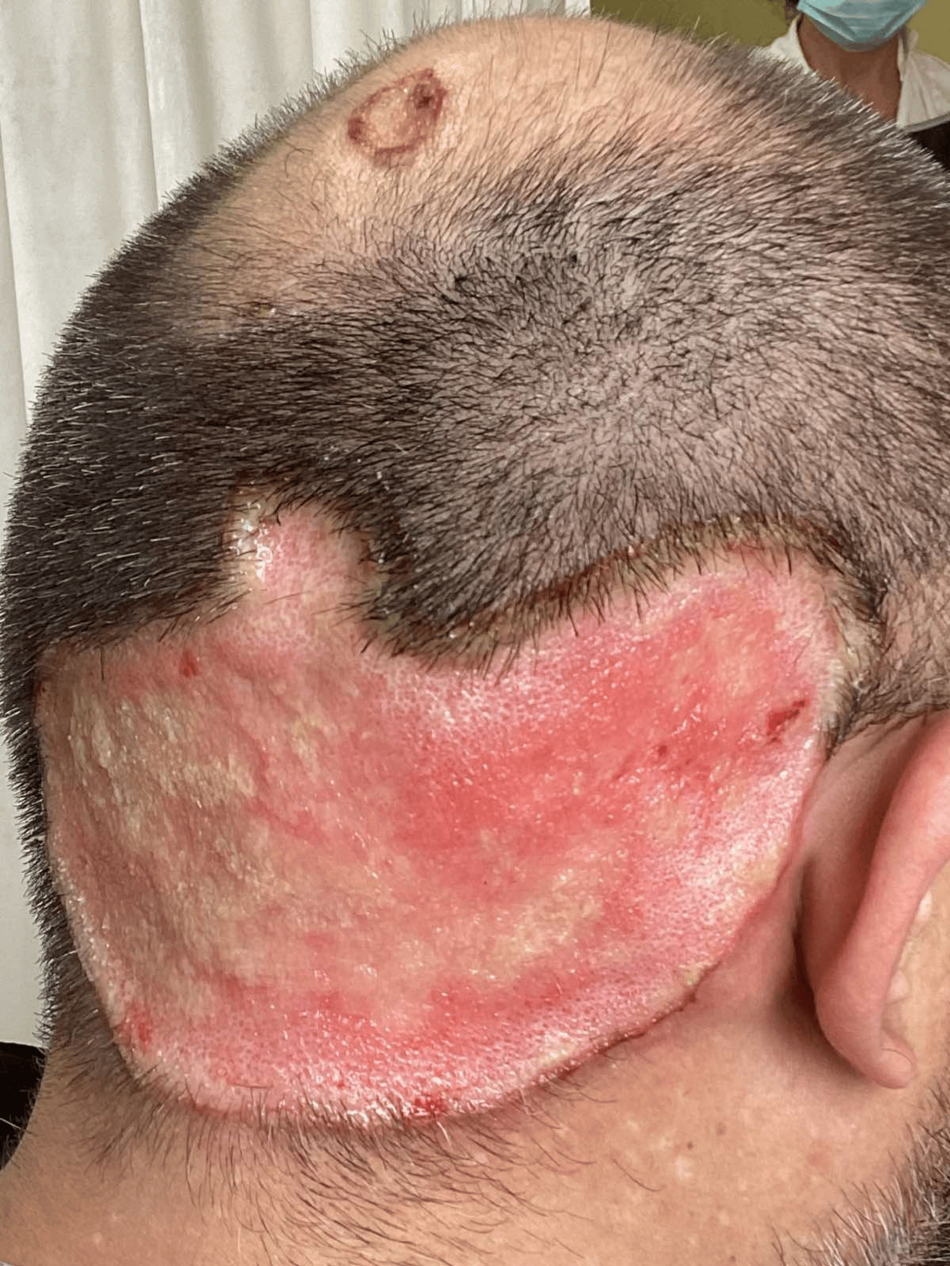
Back part of the head on the 10th day of hospitalization

A 43-year-old male, without any serious medical history, presented to our department (First Dermatology Department, Andreas Syggros Hospital for Skin Diseases, Greece) with a large, well-demarcated, ulcerated plaque with smooth margins covering almost the entire occipital area (Figure 1). According to the patient, the lesion had been caused by him within the last year, after an initial minor local injury along with a reported serious mental burden. For the past year, he had been receiving topical treatments with healing and antibiotic creams without improvement. At the time of presentation, apart from the aforementioned lesion, he complained of having a low-grade fever for the previous three days and a feeling of general malaise. The laboratory findings showed increased white blood cells, C-reactive protein (CRP), and erythrocyte sedimentation rate (ESR), indicating a possible infection and the cultures of the ulcerated lesion revealed a large amount of *Enterobacter cloacae* and *Staphylococcus aureus* colonies. The patient was hospitalized and started intravenous antibiotic treatment with ciprofloxacin 500 mg b.i.d. and clindamycin 600 mg t.i.d. along with levocetirizine 5 mg b.i.d. orally and local treatment with healing cream and antiseptic spray. After three days, the fever subsided and the laboratory tests returned within normal limits, but the patient was still expressing an urge to touch and harm the area of the lesion, along with a feeling of restlessness and insomnia. Due to this fact, bandaging was applied, and a psychiatric assessment was performed. The psychiatric history describes sleep disturbances and reduced daytime functioning, decreased appetite, insomnia, low self-esteem, fatigue, feelings of hopelessness, and reduced functionality during the six last months due to a romantic disappointment that led him to constantly occupy himself with the scalp without ever seeking psychiatric help. The psychiatric evaluation assessed skin-picking behavior as the primary cause of the skin lesion, and he was treated with sertraline 50 mg PO once daily and alprazolam 0.5 mg once daily. The final score on the Beck Depression Inventory (BDI) scale [10] was 24 associated with moderate depression and was mainly related to the symptomatology of somatic-vegetative performance complaints (consisting of the last items of the BDI scale). The patient showed a rapid, significant improvement with a reduction of his urge to pick at his wound, a better quality of sleep, and being in a calm state. He was discharged with signs of clinical improvement of the head lesion as it transitioned to the epithelialization phase (Figure 2) and scheduled to be followed up by the psychodermatology department of our hospital with main symptoms of decreased appetite, insomnia, low self-esteem, fatigue, and feelings of hopelessness.

## Discussion

Trichotillomania is characterized by a body-focused repetitive behavior in which patients direct their attention exclusively to hair pulling instead of attending to their emotions. Comorbidity includes anxiety, skin-picking disorder (SPD), substance abuse, depression, obsessive-compulsive disorder (OCD), personality disorder, eating disorders, attention-deficit hyperactivity disorder (ADHD), as well as impaired physical and mental quality of life [11]. It presents a lifetime prevalence of 1.4% to 3.1%. SPD is not an uncommon disorder, and the prognosis is better if hair pulling lasts less than 25 weeks, although the majority of the patients do not seek medical treatment [12]. Regarding the etiology, it is likely to be connected with genetic, social, and neuropsychological factors, causing negative feelings such as guilt or shame, and distraction behavior is triggered by extrinsic and proprioceptive stimuli (e.g., reading, watching TV, feeling stress, boredom, and having thoughts such as that "the hair must be symmetrical"). It can also occur in response to sexual desire, or else automatically, as a result of habit. The aforementioned may coexist simultaneously, or they may alternate at different time periods in the same patient. Some patients may keep the hairs they have pulled out to observe them later, chew them (especially their roots), and then throw them away or swallow them in the case of trichophagia [13]. Trichotillomania is defined as an obsessive-compulsive or related disorder in which patients recurrently pull out hair from any region of their body. Pharmacotherapy can be necessary, especially in adolescents and adult patients. Options include tricyclic antidepressants, selective serotonin reuptake inhibitors, and glutamate-modulating agents. Glutamate-modulating agents such as N-acetylcysteine are a good first-line option due to their significant benefits and low risk of side effects [14]. Sertraline appears to affect specific striatal-based circuits in OCD, and these changes in part could account for clinical improvement [15]. Trichotillomania causes significant subjective discomfort and impairment in the patient's functionality. Because of the fear of social exposure, enormous efforts are made to cover up areas of the body where there is visible hair loss. The patients use eyebrow pencils or wigs and avoid windy places, swimming, and generally situations from which they used to derive pleasure before the onset of the disorder. The low self-confidence presented by patients is not only due to aesthetic problems resulting from hair loss but also due to the feeling that they are hurting themselves and cannot control it [16,17]. Researchers studying the etiology of trichotillomania have found correlations to OCD based on their similarity. They report that the repetitive washing that occurs in OCD and in some patients is associated with increased activity in the basal ganglia is a normal grooming behavior that manifests itself in excess. According to behavioral theories, trichotillomania is a result of dependent and active learning [18]. Treatment options for trichotillomania include behavioral therapy (habit reversal therapy, dialectical behavioral therapy, exposure, and ritual prevention therapy), pharmacological treatment (selective serotonin reuptake inhibitors, tricyclic antidepressants, glutamate modulators, anticonvulsants, opioid antagonists, synthetic tetrahydrocannabinol), and non-invasive procedures (repetitive transcranial magnetic stimulation). It is clear that despite a recent flux of research centering on trichotillomania, substantial challenges to understanding and treating this psychological disorder still exist for researchers and clinicians. Based on this review of the literature and on our clinical experience with trichotillomania, we propose directions for future research with this underserved psychiatric group in the fields of genetic, pharmacological, neuroimaging, and psychotherapeutic research [19].

## Conclusions

Looking to the future, neuroimaging studies are ongoing to better understand the brain mechanisms involved in trichotillomania and could inform more targeted treatments. Also, genetic studies may reveal new data. Since most patients visit a dermatologist first, the vigilance of the latter is important because trichotillomania is a psychiatric disease. In addition, increased public and health professional awareness of trichotillomania is crucial to reducing stigma, improving diagnosis rates, and ensuring that individuals receive appropriate treatment. The future outlook for trichotillomania may be promising, with continued advances in research and awareness expected to improve the lives of those affected by the condition. The interplay of psychiatric disorders and dermatology is very complex yet ever-growing, and it is important for dermatologists to understand and acknowledge, as they are likely the first providers that will be alerted of these adverse conditions. It is necessary that dermatologists can identify triggering agents in a patient’s medical record, be aware of potential treatments, and know when to reach out to psychiatric colleagues for a multidisciplinary approach.
